# Avocado seed discoveries: Chemical composition, biological properties, and industrial food applications

**DOI:** 10.1016/j.fochx.2022.100507

**Published:** 2022-11-11

**Authors:** Sneh Punia Bangar, Kyle Dunno, Sanju Bala Dhull, Anil Kumar Siroha, Sushil Changan, Sajid Maqsood, Alexandru Vasile Rusu

**Affiliations:** a Department of Food, Nutrition, and Packaging Sciences, Clemson University, SC 29634, USA; b Department of Packaging Science, Rochester Institute of Technology, Rochester, NY, USA; c Department of Food Science and Technology, Chaudhary Devi Lal University, Sirsa-125055, India; d Division of Crop Physiology, Biochemistry and Post-Harvest Technology, ICAR – Central Potato Research Institute, Shimla 171001, India; e Department of Food Science, College of Agriculture and Veterinary Medicine, United Arab Emirates University, Al Ain 15551, United Arab Emirates; f Life Science Institute, University of Agricultural Sciences and Veterinary Medicine Cluj-Napoca, 400372 Cluj-Napoca, Romania; g Animal Science and Biotechnology Faculty, University of Agricultural Sciences and Veterinary Medicine Cluj-Napoca, 400372 Cluj-Napoca, Romania

**Keywords:** Avocado seed, Bioactive compounds, Phytochemical, Health-promoting effects, Industrial application

## Abstract

The processing industry discards avocado seeds, which increases production and ultimately pollutes the environment. It would be advantageous to handle these waste by-products both economically and environmentally. Avocado seeds are rich in polysaccharides, proteins, lipids, vitamins, minerals, and other bioactive substances. The nutritional and phytochemical composition of avocado seeds has been well studied and discussed.

Avocado-seed extracts also have many health-related bioactive properties, such as anti-hyperglycaemic, anticancer, anti-hypercholesterolemia, antioxidant, anti-inflammatory, and anti-neurogenerative effects are clearly demonstrated how these properties can be used to formulate or fortify food. The health-promoting properties of avocado seeds have been studied. These properties are attributed to various phytochemicals, such as acetogenin, catechin, epicatechin, procyanidin B1, estragole, etc. Additionally, items made from valorized avocado seeds that people can consume have been explored. The best applications of valorized by-products have been created for the pharmaceutical, functional food, and nutraceutical sectors while considering quality and safety. More clinical testing and product development research are required to prove the effectiveness of avocado seeds.

## Highlights

• Avocado seed are rich source of lipid, proteins, vitamins, minerals and bioactives.
• Seeds are rich in various bioactive compounds and possess diverse health benefits.
• This review summarizes nutritional and phytochemical profile of the avocado seeds.
• Avocado seeds as an ingredient in functional food is outlined in the review.

## Introduction

Avocado (*Persea americana* Mill.) crop is cultivated and highly demanded internationally because of the growing demand for fruit and food products. It is a dicotyledonous plant that belongs to the flowering plant family Lauraceae, a native of Central America and Mexico. It is mainly grown in Mexico, Saint Dominic, Peru, Indonesia, Colombia, Brazil, Kenya, Venezuela, Chile, the United States, New Zealand and South Africa (FAO, 2018). Generally, avocado seeds are discarded, considering them a waste by-products of avocado processing industries. This by-product has not been used significantly, causing serious environmental pollution (Figueroa, Borrás-Linares, Lozano-Sánchez, Quirantes-Piné, & Segura-Carretero, 2018). Effective waste by-product management would benefit from an economic and environmental perspective (Araújo et al., 2020). Seeds of avocados represent a substantial percentage (13 %–17 %) of the avocado fruit and are rich in various functional and bioactive components, namely polysaccharides, proteins, lipids, minerals, and vitamins (Melgar et al., 2018, Tremocoldi et al., 2018). Avocado seeds contain many plethoras of bioactive viz., phenolics, flavonoids, and condensed tannins. These extracts have been examined for their bioactivities, such as anti-hyperglycemic (Tremocoldi et al., 2018), anti-cancer (Lara-Marquez et al., 2020), anti-inflammation (Dabas, Elias, Ziegler, & Lambert, 2019), anti-hypercholesterolemia (Uchenna, Shori, & Baba, 2017), anti-oxidant (Soledad et al., 2021), anti-microbial (Villarreal-Lara et al., 2019), and anti-neurogenerative, with numerous traditional uses as dermatological applications. They are a good natural source of biologically active ingredients for the food, pharmaceutical, and cosmetic sectors because they contain no harmful or dangerous compounds. (Tremocoldi et al., 2018). Additionally, because of their high antioxidant potential, they prevent food oxidation, a degrading process of proteins, vitamins, carbohydrates, and lipids with reactive nitrogen and oxygen species that modifies the nutritional and sensory properties of food products (Calder & Iztapalapa, 2016). The exploring potential of seeds as a promising source of natural bioactive components can develop a novel product with added value and a safe alternative to synthetic compounds. In addition, the valorization of avocado seed residue significantly influences the environmental benefits and avocado processing industry (Saavedra et al., 2017). This review is an updated compilation of various aspects of avocado seed, such as nutritional composition, bioactive compounds, health-promoting biological activities, and its application in the food industry.

## Nutritional profile of avocado seed

The avocado seed is rich in various nutritional and bioactive compounds, especially proteins, starch, lipids, crude fiber, vitamins, minerals, and numerous phytochemicals. The nutritional profile of the avocado seed in several studies is summarized in Table 1**.**

**Table 1:** Nutritional composition of avocado seeds.

Group	Composition	References	
Proximate analysis			
Moisture Content	13.09 %	Egbuonu et al., 2018	
Dry Matter	86.91 %		
Crude Fibre	2.87 %		
Ash	3.82 %		

Sugar components (mg/g of DW)			
Hexose	1.9	Tesfay et al., 2012, Liu et al., 2002	
Glucose	5.62		
Fructose	12.93		
Sucrose	7.86–18.5		
d-Mannuheptulose	10.51–63.8		
Perseitol	12.54–88.3		
Carbohydrate (%)	64.9		

Protein	%		
Crude protein content (AOAC, 1990 method)	2.64	Egbuonu et al., 2018	
Protein content	23	Ifesan & Olorunsola, 2015	
Protein content	17.94	Arukwe et al., 2012	
Protein content	7.75	Macey et al., 2015	
Protein content	15.55	Ejiofor et al., 2018	

Lipid’s profile			
Long-chain fatty acids	(μg/g)		
Tetracosanoic acid	4.29	Báez-Magaña et al., 2019	
Nervonic acid	2.88		
Behenic acid	3.63		
Erucic acid	2.44		
Arachidic acid	2.39		
Stearic acid	5.06		
Oleic acid	5.32		
Linoleic acid	4.06		
Palmitic acid	7.1		
Myristic acid	2.49		

Fatty acid derivatives (aliphatic acetogenins)			
Avocatins	32.28	Báez-Magaña et al., 2019	
Polyhydroxy fatty acids	24.26		
Pahuatins	4.26		
Persins	10.12		

Minerals	mg/100 g		
Calcium	0.82	Ifesan & Olorunsola, 2015	
Potassium	4.16		
Phophorus	0.09		
Zinc	0.18		
Sodium	1.41		
Iron	0.31	Arukwe et al., 2012	
Copper	0.98		

Vitamins	mg/100 g		
Vitamin A	10	Seed et al., 2017	
Thiamin	0.33		
Riboflavin	0.29		
Niacin	0.06		
Ascorbic acid	97.8		
Vitamin E	0.12		

### Carbohydrates

Among all the macromolecules found in avocado seeds, carbohydrates are said to make up a significant portion (64.9 %). Starch makes up 91.2 % of the total carbohydrates in avocado seeds (Tesfaye et al., 2018). It has been found that plant-based polysaccharide fractions contain a variety of biological activities (Bangar, Ashogbon, Lorenzo, Phimolsiripol, & Chaudhary, 2022). The two C7 sugars, namely perseitol (88.3 mg/g) and d-mannoheptulose (63.8 mg/g), were abundant in avocado seeds (Liu, Sievert, Lu Arpaia, & Madore, 2002). The dominance of these C7 sugars in avocado seeds indicates their importance in these tissues. These sugars might have a role as transport and storage sugars in avocados. Tesfay, Bertling, and Bower (2011) concluded that the abundance of perseitol, at physiological maturity, among all sugars in the avocado cotyledons indicates their role as a C7 carbon storage compound. Liu et al. (2002) reported the carbohydrate profile of avocado seed as 246.1 (starch), 18.5 (sucrose), 1.9 (hexose), 63.8 (d-mannoheptulose), and 88.3 (perseitol) mg/g of dry weight (DW). The quantity of C7 sugar found was 36.3 % of the total sugars in the avocado seed. Similarly, another study reported various sugars, including fructose (12.93), glucose (5.62), sucrose (7.86), d-mannoheptulose (10.51), and perseitol (12.54 mg/g of DW) (Tesfay, Bertling, Bower, & Lovatt, 2012) present in avocado seeds.

### Lipids

Plant-derived lipids are mostly used for food and non-food industrial utilization. Takenaga, Matsuyama, Abe, Torii, and Itoh (2008) investigated the fatty acids and lipid profile of avocado seeds from 3 different cultivars: Bacon, Fuerte, and Hass. They reported total lipid (TL) content of1.1 %–1.6 % in avocado seeds. Further analysis of TL using thin-layer chromatography revealed the presence of neutral lipid, glycolipid (GL), and phospholipid (PL) as 77.1–80.3, 12–13.2, and 7.4–10.9 % of TL, respectively. Authors reported GL composition as 17.5–18.6 (acylsterylglucoside), 56.3–57.7 (monogalactosyl-diacylglycerol), 10.1–10.8 (sterylglucoside), 9.8–10.7 (cerebroside), 1.7–2.0 (digalactosyl-diacylglycerol) and 1.9–2.4 % (others) of total GL. While PL composition contains 14.5–17.6 (phosphatidic acid), 30.7–31.9 (phosphatidyl ethanolamine), 10.5–13.4 (phosphatidylglycerol), 28.9–31.4 (phosphatidylcholine), 3.6–4.2 (phosphatidylinositol) and 6.3–6.9 % (others) of total PL. An investigation of the fatty acid profile of avocado seeds displayed that linoleic acid is present in the highest amount (35 %–38 %), followed by oleic acid (22 %–24 %) and palmitic acid (17 %–19 %). Similarly, Báez-Magaña, Ochoa-Zarzosa, Alva-Murillo, Salgado-Garciglia, and López-Meza (2019) performed fatty acid profiling of the lipid-rich extract of avocado seeds by GC–MS. They reported fatty acids, including palmitic (7.1 μg/g), nervonic (2.88 μg/g), arachidic (2.39 μg/g), linoleic (4.06 μg/g), oleic (5.32 μg/g), stearic (5.06 μg/g), myristic (2.49 μg/g), erucic (3.63 μg/g) andtetracosanoic acid (4.29 μg/g), and their derivatives such as avocations (32.28 μg/g), persins (10.12 μg/g), polyhydroxy fatty acids (24.26 μg/g), and pahuatins (4.26 μg/g). These results concluded that avocado seeds extract is abundant in fatty acids (particularly oleic, linoleic, and palmitic acid) and derivatives, viz., acetogenins, pahuatins, persins, avocatins, or fatty acid alcohols.

### Protein

Protein is a major component among various macromolecules in avocado seeds (Egbuonu, Opara, Onyeabo, & Uchenna, 2018). Proteins are large, complex molecules made of amino acids that play a key role in growth and development, cell signaling, enzyme regulation, and biocatalysts. Due to the increased need for nutritionally superior food, plant-based nutrients, especially protein, have gained attention. Thus, much emphasis has been given to finding sustainable alternative nutritionally dense food sources (Lonnie et al., 2018). Various studies reported protein content in avocado seeds as 23 % (Ifesan & Olorunsola, 2015), 17.94 % (Arukwe et al., 2012), 15.55 % (Ejiofor, Ezeagu, Ayoola, & Umera, 2018), 7.75 % (Mahawan, Francia, Tenorio, Gomez, & Bronce, 2015), and 2.64 % (Egbuonu et al., 2018). Thus, the substantial amount of nutrients in avocado seeds, including carbohydrate, protein, and dietary fibers, could warrant their utilization in human supplements (Ejiofor et al., 2018). There are limited research reports available with regard to the quantified amino acids and protein in the avocado seeds; therefore, more focus is required to unearth its amino acid and protein profiles.

### Minerals and vitamins

The avocado seeds are a rich source of various minerals, namely phosphorus (P), calcium (Ca), potassium (K), iron (Fe), sodium (Na), zinc (Zn), copper (Cu), cobalt (Co), and lead (Pb), and vitamins including vitamin A, thiamine (B1), riboflavin (B2), niacin (B3), Vitamin C and vitamin E. Ifesan and Olorunsola (2015) found the concentration of various minerals, namely, P, Ca, Na, and Zn as 4.16, 0.09, 0.82, 1.41, and 0.18 mg per 100 g of the avocado seed, respectively. The minerals in avocado seeds make them a preferable choice for animal feed and human nutrition to fulfill micronutrient deficiency (Justina, Olukemi, Ajayi, & Adegoke, 2016). Egbuonu, Opara, Atasie, and Mbah (2017) observed the concentration of various vitamins as 10 (A), 0.33 (B1), 0.29 (B2), 0.06 (C), and 0.12 (E) mg per 100 g of the avocado seed. The vitamins A, C, and E in the avocado seed may improve the health of the immune system, vision, and blood vessels. In contrast, vitamin B displays a major role in cognitive function stimulation, nerve relaxation, and improving blood circulation.

### Bioactive compounds in avocado seeds

Recently, numerous research and reviews articles on the utilization of by-products of horticultural crops showed that phytochemicals and their health-promoting activities could boost their use in the preparation of innovative foods (Bangar et al., 2022, Punia and Kumar, 2021). This will improve the overall profitability of the farmers and reduce the cost of disposal of the by-products. Avocado seeds contain severalfold phenolics compared to popular antioxidant sources such as raw blueberry (Wang, Bostic, & Gu, 2010). It constitutes phenolics from five groups viz., procyanidins, catechins, flavonols, hydroxycinnamic, and hydroxybenzoic acids (Rodríguez-Carpena, Morcuende, Andrade, Kylli, & Estévez, 2011). Further, Kosińska et al. (2012) reported 9.5 and 13.04 mg CE/g dry weight (DW) in Hass and Shephard varieties of avocado. In contrast, Soong and Barlow (2004) stated relatively high levels of 88.2 mg of GAE/g of DW. The variation in the bioactive profile is attributed to the variety, soil type, agronomic conditions, and post-harvest handling of the fruits (Kosińska et al., 2012). Specific phenolics in avocado seeds were identified using UV spectra characteristics and retention times, and HPLC-ESI-MS was employed for the structural confirmation. Catechin/epicatechin gallate, 3-*O*-caffeoylquinic acid, procyanidin trimer A (II), 3-*O*-p-coumaroylquinic acid procyanidin trimer A (I), were found in the concentration presented in Table 2 **(**Kosińska et al., 2012). In another study, phenolic compounds in Hass and Fuerte variety were evaluated using chromatographic analysis. The authors identified four phenolic compounds, namely *trans*-5-O-caffeoyl-d-quinic acid, procyanidin B1, catechin, epicatechin, and the concentrations of the respective compounds are shown in Table 2. The volatile compounds of the seed extracts were investigated and showed esters of fatty acids and their derivatives and isoprenoid derivatives (Soledad et al., 2021). Under the terpenoid and phenylpropanoid compounds category, seven compounds were identified: estragole, isoestragole, cubebene, α-cubebene, α- germacrene α-farnesene, and caryophyllene. Another important component of the lipid fraction of avocado seeds is polyhydroxylated fatty alcohol (PHFA) derivatives. Acetogenins (type of PHFA) originated from fatty alcohols with unsaturated aliphatic chains, commonly acylated. The concentration of total acetogenins varied between 1090 and 8330 μg/g DW in avocado seed among 22 cultivars. Acetogenins viz., persenone A & B, AcO-avocadene contributed the maximum to the acetogenin profile of the avocado seeds, followed by persenone C, AcO-avocadenyne, persin, and persediene (Rodríguez-López, Hernández-Brenes, & de la Garza, 2015). Alkaloids, phytosterols, and tocopherols are other minor components in avocado seeds.

**Table 2:** Bioactive compounds associated with avocado seeds.

Source	Compound	Cultivar and concentration	References
Total phenolic content	–	Hass: 9510 and Shephard: 13040 μg/g dw	Kosińska et al. (2012)
Total phenolic content	–	Hass: 57,300 and Fuerte: 59200 μg/g dw	Tremocoldi et al. (2018)
Phenolic compounds and its derivatives			
Phenolic acids			
Queensland, Australia	3-O-caffeoylquinic acid	Hass: 57.5 and Shephard: 53.5 μg/g dw	Kosińska et al. (2012)
Queensland, Australia	3-O-p- coumaroylquinic acid	Hass:13.6 and Shepard: 8.1 μg/g dw	Kosińska et al. (2012)
Jaguacy Avocado Brasil Bauru, SP, Brazil	*trans*-5-O-caffeoyl-d-quinic acid	Hass: 1630 and Fuerte: 5740 μg/g dw	Tremocoldi et al. (2018)
Flavonoids			
Queensland, Australia	Catechin/ epicatechin gallate	Hass: 152.8 and Shephard: 105.4 μg/g dw	Kosińska et al. (2012)
Jaguacy Avocado Brasil Bauru, SP, Brazil	Epicatechin	Hass: 10,270 and Fuerte: 11060 μg/g dw	Tremocoldi et al. (2018)
Jaguacy Avocado Brasil Bauru, SP, Brazil	Catechin	Hass: 3640 and Fuerte: 8130 μg/g dw	Tremocoldi et al. (2018)
Procyanidins			
Queensland, Australia; Level of ripening: ready-to-eat ripeness	Procyanidin trimer A (I)	Hass: 81.7 and Shepard: 98.9 μg/g dw	Kosińska et al. (2012)
Queensland, Australia; Level of ripening: ready-to-eat ripeness	Procyanidin trimer A (II)	Hass: 89.3 and Shepard: 73 μg/g dw	Kosińska et al. (2012)
Jaguacy Avocado Brasil Bauru, SP, Brazil	Procyanidin B1	Hass: 48,380 and Fuerte: 28340 μg/g dw	Tremocoldi et al. (2018)
Polyhydroxylated fatty alcohol derivatives			
Fundacion Sanchez Colın – CICTAMEX, Coatepec Harinas, Estado de Mexico, Mexico	Acetogenins	1090 to 8330 μg/g dw in 22 cultivars of avocado	Rodríguez-López et al. (2015)
Fundacion Sanchez Colın – CICTAMEX, Coatepec Harinas, Estado de Mexico, Mexico	Persin	0–300 μg/g dw in 22 cultivars of avocado	Rodríguez-López et al. (2015)
Alkaloids			
Botanical garden in Akure metropolis, Nigeria	Hyoscyamine 0.6	600 μg/g dw	Oboh et al. (2016)
	Atropine	460 μg/g dw	
	Scopolamine	240 μg/g dw	
	Norhyoscyamine	40 μg/g dw	
	Solanidine	41 μg/g dw	
	Solanin	40 μg/g dw	
	Solasoline	80 μg/g dw	
Phytosterols			
Local market in Egypt	Campesterol	–	Alkhalaf et al. (2019)
	Stigmasterol	1.11 %	
	β-sitosterol	2 %	
–	5α-colestane	49.77 μg/g dw	Barrera López and Arrubla Vélez (2017)
	Stigmasterol	19.17 μg/g dw	

### Health-related bioactive properties of avocado seed extracts

**Fig. 1:**
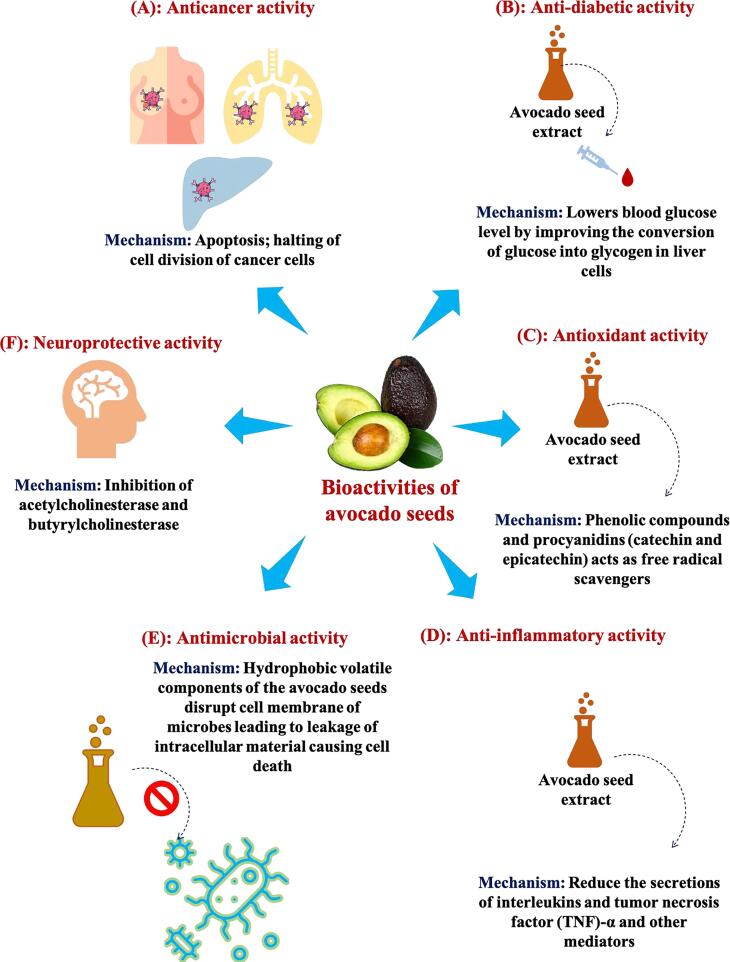
Biological activities of the avocado seed extract.

Due to their importance in human health, the separation and identification of functional components from natural resources have become the main research focus of the food, nutraceutical, and pharmaceutical industries. This is because these components play a role in various biological and health-promoting processes in the human body. Avocado seeds are high in phytochemicals and are utilized for medicinal purposes. The bioactivities of avocado seed extracts will be discussed in the sections below. An illustration showing various bioactivities of the avocado seed extract is presented in Fig. 1**.**

### Anticancer activity

Globally, cancer has become a serious health issue, with the global cancer burden increasing to 18.1 million with 9.6 million deaths (GLOBOCAN, 2018). Cancer is characterized by the growth and multiplication of abnormal cells that invade neighboring tissues and spread outward (Zheng, Zhang, & Zeng, 2016). Synthetic anti-tumor medications have been found in clinical research to have possible therapeutic results but substantial toxicity to normal cells, posing a threat to human health. Due to its safety and immune-enhancing effect in humans, plant sources are gaining interest as anti-tumor medicines with lower toxicity. Avocado seeds and their biologically active components exhibited anti-cancer potential in human and animal cell lines, including prostate and lung cancer (Dabas et al., 2019), breast cancer (Dabas et al., 2019, Widiyastuti et al., 2018), colon cancer cells (Alkhalaf et al., 2019, Dabas et al., 2019), and hepatocellular carcinoma (Alkhalaf et al., 2019). Polyphenols from avocado seeds can inhibit human prostate cancer cells (LNCaP), breast cancer cells (MCF7), lung cancer cells (H1299), and colon cancer cells (HT29) with inhibition rates of 19, 19.1, 67.6, and 132.2 μg*/*mL in a dose-dependent manner (Dabas et al., 2019).

The authors explained that avocado seed extracts induced G_0_/G_1_ cell cycle arrest via downregulating cyclin D_1_ and E_2_ expression in prostate cancer cells. Further, similar results were shown by Lee, Yu, Lee, and Lee (2008) in breast cancer cell lines (MDA-MB-231) by methanolic extracts of avocado seeds. Seed extracts (0.1 mg/mL) increased activation of caspase-3 and caspase-3 target protein, poly (ADP-ribose) polymerase (PARP), resulting in apoptosis. Ethanolic extracts of avocado seeds induced apoptosis in Jurkat lymphoblastic leukemia cells in an oxidative stress-dependent manner through depolarization of the mitochondrial membrane, activating protease caspase-3, and transcription factor p53, and predominancy of apoptosis-inducing factor (Bonilla-Porras, Salazar-Ospina, Jimenez-Del-Rio, Pereañez-Jimenez, & Velez-Pardo, 2014). Avocado seeds can inhibit the proliferation of immortalized HaCaT keratinocytes, which could be due to proanthocyanidins B1, proanthocyanidins B2, and A-type trimer (Ramos-Jerz, Villanueva, Jerz, Winterhalter, & Deters, 2013).

Triterpenoid, an important secondary metabolite in avocado seeds, has anticancer activity (Iskandar, Novriyani, Damayanti, Afriani, Sukmawaty, Iqraini, & Razak, 2019). These secondary metabolites disrupt the membrane permeability of the mitochondrial cell wall, resulting in cell necrosis. It has been reported that triterpenoids have cytotoxic activity for lung cancer cells (A549), gastric cancer cells (SGC-7901), breast cancer cells (MCF-7), liver cancer cells (HepG2), and colon cancer cells (HCT15) (Hu et al., 2014). Further, ethanolic extract of avocado seeds triterpenoids displayed significant cytotoxic activity against Vero, human breast cancer cells (MCF-7), and human liver carcinoma cells (HepG2). *In vitro* 3-(4,5-dimethylthiazol-2-yl)-2,5-diphenyl tetrazolium bromide (MTT) assay displayed that triterpenoid of avocado seeds have the potential to inhibit proliferation of MCF-7 and HepG2 having the IC_50_ values of 62 and 12 mg/mL, respectively (Abubakar, Achmadi, & Suparto, 2017). Ethanolic extracts of phenolic compounds, alkaloids, glycosides, and saponins were reported to have a cytotoxic effect on breast cancer (T47D) cell lines with IC_50_ values of 107 μg/mL (Kristanty, Suriawati, & Sulistiyo, 2014).

Lipidic extracts of avocado seeds were targeted for anticancer action on the HCT116 and HepG2 cancer cells. Authors described that seed lipids at a concentration of 100 µL exhibited an inhibitory percentage of 65 and 58 % in HCT116 and HepG2 cancer cell lines compared to avocado fruit lipids (Alkhalaf et al., 2019). Ethnopharmacological studies of Widiyastuti et al. (2018) reported cytotoxic and apoptosis effects of avocado seeds on MCF-7 cell lines. The authors examined cytotoxic activity by MTT assay and apoptosis by flow cytometric analysis. The cytotoxic test revealed the potent cytotoxicity of chloroform extract on MCF-7 cancer cell lines with an IC_50_ concentration of 94.9 μg/mL.

Moreover, increased cytotoxicity with IC_50_ of 34.5 and 66.0 μg/mL was observed for methanol-soluble and non-soluble forms. Flow cytometry study concluded that methanolic fraction induced apoptosis by modulating sub-G1 phase arrest in MCF-7 cells. The lipidic extract of avocado seeds also has a cytotoxic effect on colorectal cancer. The avocatins and polyhydroxylated fatty alcohols in avocado seeds are associated with the possible cytotoxic reaction on Caco-2 cells (Lara-Marquez et al., 2020). These compounds induced apoptosis by activating caspases 8 and 9. Extracts can induce loss of mitochondrial membrane potential, inhibit fatty acid oxidation, and increase the superoxide anion (O_2_^−^) and mitochondrial reactive oxygen species (ROS). Additionally, lipidic extracts encouraged the release of cytokines IL-6, IL-8, and IL-10; but inhibited IL-1β secretion.

### Antidiabetic activity

Diabetes mellitus is a common genetic disorder caused by the impairment of insulin secretion and its deficiency. The International Diabetes Federation (IDF) reported that diabetes mellitus had reached epidemic levels worldwide. Currently, 463 million people and about 10 % (USD 760 billion) of global health expenditures are on diabetes (IDF, 2019). Chronic hyperglycemia is caused by insulin insufficiency, disturbing carbohydrate, protein, and lipid metabolism. Type 2 diabetes can be delayed and managed by altering one's lifestyle and developing good habits. Natural products with anti-diabetic properties could be a viable option to treat diabetes with minimum adverse effects (Zhao et al., 2018). Avocado seed help in treating type 2 diabetes by targeting peroxisome proliferator-activated receptor-gamma in the same way as an anti-diabetic drug (thiazolinediones) (Dabas, Shegog, Ziegler, & Lambert, 2013). Avocado seeds (2 %–8%) were added to a high-sugar diet and given to spontaneously hypertensive rats, which had an anti-diabetic and lipid-lowering impact by lowering blood glucose and cholesterol. The blood-glucose-lowering effect was attributed to bioactive compounds that assist in depositing glucose into the glycogen in the liver cells (Uchenna et al., 2017). In alloxan-induced diabetic rats, treatment of 300 or 600 mg/kg body weight avocado seed extract lowered glycemia (>70 %) and restored damage to pancreatic islet cells (Edem, Ekanem, & Ebong, 2009).

Supplementation of 40 g/L of hot aqueous avocado seed extracts and glibenclamide (5 mg/kg) to alloxan-induced Wistar albino rats significantly decreased the blood glucose of diabetic rats. They observed that the reference drug glibenclamide provided the highest response (58.9 %) on day 14, equivalent to the reaction of 40 g/L avocado seed extract on day 21 (Ezejiofor, Okorie, & Orisakwe, 2013). According to pancreas histology, the normal control rats had intact pancreatic islets and exocrine cells. Alloxan-induced diabetes rats (diabetic control rats) showed reduced islet cells and necrosis regions. Compared to the untreated alloxan-induced diabetic rats, diabetic rats treated with the 20 g/L extracts showed tiny, maintained islet cells. The studies above have revealed that avocado seeds extract may have anti-diabetic characteristics, indicating that more study is needed.

### Antioxidant activity

Free radicals are generated due to oxidative stress and autoxidation of human lipids and lipoproteins, which are linked to diabetes, cardiovascular disease, respiratory disease, cancer, neurodegenerative and many other diseases (Punia et al., 2020, Dhull et al., 2020). An interest in using natural plant antioxidants, including polyphenols, flavonoids, and alkaloids, is increasing daily to solve these health problems (Dhull, Kaur et al., 2020; Bangar et al., 2022c). These compounds can quench free radicals, scavenge free oxygen and chelate catalytic metals (Kaur, Dhull, Sandhu, Salar, & Purewal, 2018), which have shown promising potential in reducing oxidative stress, preventing several diseases, maintaining health, and delaying the aging process. Avocado seed displays in vitro antioxidant potential by stabilizing peroxyl radicals and superoxide anions and DPPH and ABTS, ferric reducing power, inhibiting the β-carotene blanching and development thiobarbituric acid reactive substances (Tremocoldi et al., 2018). Colored avocado seed extracts displayed oxygen radical absorbance capacity (ORAC) of 2012 Trolox equivalents/mg, and electron paramagnetic resonance spectroscopy assay observed radical scavenging potential of seed extracts with EC_50_ of 42.1 μg/mL (Dabas et al., 2019). A dose of 0.75 % avocado seed extracts causes an 80 % delay in oxidation as measured by oxidation induction time (Segovia, Hidalgo, Villasante, Ramis, & Almajano, 2018).

Aqueous extracts of avocado seeds exhibit antioxidant potential and can prevent radical-induced oxidative damage (Oboh et al., 2016). The authors induced rat brains with Fe^2+^ and sodium nitroprusside (SNP) solutions. They observed an increase in thiobarbiturate reactive species (TBARS) level resulting in oxidative damage caused by free radicals by Fe^2+^ and SNP. Furthermore, avocado seed extract reported decreased TBARS levels in Fe^2+^ and SNP-induced lipid peroxidation due to the synergic effect of phenolic components and saponins of seeds. In avocado seeds, phenolic components and procyanidins (catechin and epicatechin) contribute 38 % antioxidant activities of whole avocado fruit (Wang et al., 2010). Ethanolic extracts of Hass and Fuerte avocado seeds possessed many phenolic components (Tremocoldi et al., 2018). The reported antioxidant potential of 1175.1 and 1881.4 μmol Fe^2+^/g for Hass and Fuerte peel extracts. They reported that epicatechin and catechin in seeds could stabilize peroxyl radicals (ROO^.^) and superoxide anions (O^2.^). Interestingly, catechin had 1.3, 2.5, and 1.6 folds better-stabilizing activity to stabilize ROO^.^, O^2.^, and hypochlorous reactive species than epicatechin. Lyophilized avocado seed power was added in oil in water emulsion and beef meat burger to evaluate the delay in oxidation (Gómez, Sánchez, Iradi, Azman, & Almajano, 2014). They observed oxidation inhibition of 30 % (pure extracts) and 60 % (extract + egg albumin) in emulsion and 90 % inhibition of TBAR substances in meat burgers. The authors suggested that avocado seeds could be used in meat to increase shelf life.

The volatile or lipophilic chemical profile of avocado seeds indicates their potential application as an antioxidant additive (Soledad et al., 2021). Acetone and ethanolic extracts of avocado seeds total phenolic content of 30.80 and 30.25 GAE/100 g, respectively, and DPPH inhibition of 212.75 and 183.75 mg Trolox/100 g, respectively. Also, acetone extract of avocado seeds exhibited a higher power reduction of 56.35 ascorbic acid equivalents (AAE)/100 g than ethanol extract (45.05 g AAE/100 g). Therefore, it is suggested that avocado seeds have potential application as an antioxidant additive in food products.

### Anti-neurogenerative

Alzheimer's disease (AD) is a brain disorder characterized by the gradual degeneration of nerve cells, which leads to deficits in cognitive ability (Oboh et al., 2016). A cholinergic hypothesis states that acetylcholinesterase (AChE) and butyrylcholinesterase (BChE) catalyze the breakdown of acetylcholine into choline and acetate groups. Several studies indicate that 40–90 % of AD patients had reduced AChE activity and increased BChE activity (Brimijoin, 1983). Reducing acetylcholine levels may inhibit brain transmission in Alzheimer's patients (Ahmed, Ghalib, Sasikala, & Ahmed, 2013). Therefore, restoring acetylcholine by inhibiting AChE and BChE with phytoconstituents from plants is the modern method for treating Alzheimer's disease and neurodegenerative illnesses. Avocado seed aqueous extracts inhibited the AChE and BChE enzymes with IC50 values of 27.93 and 30.08 mg/mL, respectively (Oboh et al., 2016). Inhibition of both enzymes by phenolic compounds such as caffeic acid in avocado seeds inhibits the breakdown of butyrylcholine and acetylcholine in the brain neurons, resulting in increased levels of these neurotransmitters at the synaptic clefts. This ultimately improves the communication between the nerve cells and helps to manage neurodegenerative ailments such as AD. Various bioactivities of the avocado seed extracts are presented in Table 3**.**

**Table 3:** Health-related bioactive properties of extracted compounds from avocado seeds.

Region		Extraction method/solvent used	Compounds/component	Model	*In vitro*/*In vivo*	Target mechanism	Key findings	References
Anticancer activity								
	Mexico	Lipidic extract	Avocatins, polyhydroxylated fatty alcohols and saturated long-chain fatty acids	Colon cancer cell line Caco-2	*In vitro*	(+) Caspases 8 (+) Caspases 9 (+) Cytokines IL-6 (+) IL-8 (+) IL-10(-) IL-1β	-	Lara-Marquez et al. (2020)
	USA	Methanol	Polyphenols	–	*In vitro*	(-) Cyclin D1(-) Cyclin E2 (+) Caspase 3 (+) PARP	Avocado seed extracts could be a functional source of anticancer compounds	Dabas et al. (2019)
	Saudi Arabia	Methanol/chloroform	Lipids	HepG2 and HCT11 cancer cell lines	*In vitro*	–	–	Alkhalaf et al. (2019)
	Vietnam	Ethanol	–	–	*In vitro*	–	–	Vo and Le (2019)
	Indonesia	Chloroform and methanol	–	–		(+) Apoptosis	–	Widiyastuti et al. (2018)
	Indonesia	Ethanol	Triterpenoid	–	*In vitro*	–	Avocado seed triterpenoids exhibit cytotoxic activity with low IC50 value	Abubakar et al. (2017)
	Colombia	Ethanol	–	–	*In vitro*	(+) Transcription factor p53 (+) Protease caspase-3 (+) AIF	Avocado seeds function as a pro-apoptotic component	Bonilla-Porras et al. (2014)
	Jakarta	Ethanol	Phenolic compounds, alkaloids, saponins	T-47D breast cancer cell line	*In vitro*	–	–	Kristanty et al. (2014)
	Germany	Methanol	Proanthocyanidins B1, Proanthocyanidins B2 and A-type trimer	–	*–*	–	–	Ramos-Jerz et al. (2013)
	Korea	Methanol	Polyphenols	–	*In vitro*	(+)Caspase 3 (+) PARP (+) apoptosis	–	Lee et al. (2008)

*Antidiabetic activity*								
	Brazil	Ethanol	Phenolic compounds	–	*In vitro*	_–_	To stabilize peroxyl radicals (ROO.), superoxide anions (O2.) and hypochlorous reactive species.	Tremocoldi et al. (2018)
	Saudi Arabia		seed-supplemented diet	Anti-hyperglycemia and Anti-hypercholesteremia	*In vitro*	_–_	Avocado seeds extract Improved carbohydrate and lipid metabolism.	Uchenna et al. (2017)
	Nigeria	Hot water	–	–	*In vivo*	_–_	Seeds have anti-diabetic and protective effects on some rat tissues such as the pancreas, kidneys, and liver.	Ezejiofor et al. (2013)

*Antioxidant activity*								
	Mexico	Acetone/ethanol	Lipophilic compounds	Antioxidant potntial	*In vitro*	(+) Phenolic compounds (+) DPPH inhibition (+) Reducing power	Avocado seeds have potential application as antioxidant additive.	Soledad et al. (2021)
	Spain	Methanol; Ethanol/water	Catechin epicatechin	Radical scavenging activity	*In vivo*	(-) oxidation	Avocado seed extracts are effective as a natural antioxidant	Segovia et al. (2018)
	Nigeria	Aqueous extracts	Phenolic compounds and alkaloids	–	*In vivo*	_–_		Oboh et al.(2015)

Anti-neurogenerative activity								
	Nigeria	Aqueous extracts	Phenolic compounds and alkaloids		*In vivo*	(-) AChE(-) BChE	Avocado seed extracts may serve as a cheap therapeutic drug for preventing/treating AD.	Oboh et al. (2015)

Anti-inflammation activity								
	USA	Methanol	Polyphenols	RAW264.7 cells	*In vitro*	(-) IL-6(-) TNF-α(-) IL-1β(-) NO	Avocado seeds exhibited anti-inflammatory compounds which could be as functional food ingredients.	Dabas et al. (2019)
	Saudi Arabia	Methanol/chloroform	Lipids		*In vitro*	–		Alkhalaf et al. (2019)
	Brajil	Ethanol	Phenolic compounds	RAW264.7 cells	*In vitro*	(-) TNFα (+) Nitric oxide		Tremocoldi et al. (2018)
	Indonesia	Infusion and methanol						Kristanti et al. (2017)

Antimicrobial activity								
	Mexico	Acetone/ethanol	Lipophilic compounds	*Staphylococcu aureus* and Salmonella* enterica* serovar typhimurium	*In vitro*	(-) gram positive bacteria(-) gram negative bacteria	Maximum microbial reductions of 4 and 1.8 log cycles for Staphylococcus aureus and Salmonella enterica serovar Typhimurium	Soledad et al. (2021)
	USA	Dichloromethane	Acetogenins		*In vitro*	(-) gram positive bacteria		Villarreal-Lara et al. (2019)
	Mexico	Heptane:Methanol and Heptane: ethanol:methanol:water	Acetogenins	*Clostridium sporogenes* PA 3679	*In vitro*	(-) *C. sporogenes*	Avocado seeds as a potential source of functional compounds with anti-inflammatory activity	Pacheco et al. (2017)
	Mexico	Heptane:Methanol and Heptane: ethanol:methanol:water	Acetogenin	*Listeria monocytogenes* (ATCC 35152)	*In vitro*	(-) Listeria monocytogenes	An anti-listeral agent	Salinas-Salazar et al. (2017)

IL-6-Interleukin 6; IL-1β-interleukin-1β; No-Nitric oxide; TNF-α-tumor necrosis factor alpha.

### Anti-inflammatory

The inflammatory response is a defense response of an individual against invaders. It involves various chemical mediators capable of triggering vascular changes, such as plasma protein extravasation and defense cell recruitment. (Tremocoldi et al., 2018). Many immune cells involved in the inflammatory response, such as neutrophils, macrophages, and phagocytes, secrete inflammatory mediators such as nitric oxide (NO), interleukins, and TNF-, as well as inflammatory proteins such as nitric oxide synthase (NOS) and cyclooxygenase (COX)-2 in response to exogenous stimulation (Hyung, Ahn, Kim, Kim, & Je, 2016). Excessive inflammatory mediator release due to inflammatory responses has been linked to atherosclerosis, diabetes, tumor growth, immunological illness, and other inflammatory diseases. A novel glycosylated benzotropolone-containing polyphenol was identified in colored avocado seed extracts (Hatzakis, Mazzola, Shegog, Ziegler, & Lambert, 2019). Many studies have reported the anti-inflammatory effect of benzotropolone-containing natural products. At the dose of 6 µg/mL, avocado seeds extract reduced the secretions of IL-1β and tumor necrosis factor (TNF)-α in lipopolysaccharide-stimulated RAW264.7 cells. Avocado seed extracts at a concentration of above 5 µg/mL reduced NO production resulting in reduced inducible nitric oxide synthase (iNOS) expressions. Similar findings were reported by (Tremocoldi et al., 2018) for avocado seed extracts of Hass and Fuerta cultivars, which can inhibit TNFα and produce NO in lipopolysaccharides-stimulated RAW 264.7 macrophage culture (Tremocoldi et al., 2018). Kristanti, Simanjuntak, Dewi, Tianri, and Hendra (2017) demonstrated the anti-inflammatory activities of infusion (0.67 g/kg BW) and methanolic (3.33 g/kg BW) extract of avocado seed in carrageenan-induced paw edema in mice. They observed a decrease in area under curve values and percentage inhibition of inflammation results in the decreased thickness of paw edema on the test animals’ paws.

### Anti-microbial activity

Numerous studies have been conducted to find natural alternatives to the synthetic antimicrobial used in food, medicine, and pharmaceuticals. These efforts have been made for consumers' concerns regarding the safety of synthetic chemical products. Many scientists claimed the potentiality of avocado seed to control human food-borne pathogenic bacteria and spoilage microbes. Leite et al., 2009, Idris et al., 2009 concluded that avocado seeds organic extracts inhibited Candida spp., *Cryptococcus neoformans,* and *Malassezia pachydermatis* and bacteria including *S. aureus, S. pyogenes, C. ulcerans, C. albicans, E. coli, and S. typhi*. Further, methanolic and chloroform extract of avocado seed exhibited antifungal potential against *Cryptococcus neoformans* with IC50 value of less than 8 μg/mL and 8.211 μg/mL, and petroleum ether extracts exhibited inhibition activity against *S. aureus*, IC50 8.7 μg/mL (Falodun et al., 2014). Jiménez-Arellanes, Luna-Herrera, Ruiz-Nicolás, Cornejo-Garrido, Tapia, and Yépez-Mulia (2013) also observed anti-parasital activity of seeds for *E. histolytica,* and *G. lamblia*.

Avocado seeds contain fatty acid derivatives with antimicrobial potential called acetogenins. The first report on the antilisteral potential of avocado acetogenins was conducted by Salinas-Salazar et al. (2017). They identified AcO-avocadene, persediene, persenone C, persenone A, persin, and persenone B in avocado seeds and considered persenone C, persenone A, and AcO-avocadenyne as the most powerful acetogenin. The acetogenin extracts showed a minimum inhibitory concentration of 7.8 mg/L and a bactericidal activity due to an enhancement in membrane permeability resulting in cell lysis. Further, they added that antilisteral activity is a combined result of the *trans*-enone feature and the number of unsaturated molecules in the aliphatic chain.

In another study, acetogenins (AcO-avocadene, AcO-avocadenyne, persediene, persenone A, persenone B, persenone C, and others), naturally occurring lipidic molecules of avocado seeds were evaluated to control growth and endospore germination of *Clostridium sporogenes* PA 3679 (ATCC 7955)in carrot puree under high hydrostatic pressure (HHP) (300–600 MPa), time (3–6 min), temperature (25–120 °C) and salt (1 %–3%). The authors reported that AcO-avocadene exhibited the highest antimicrobial activity, whereas the extract was resistant to high temperature, HHP, and salt, with greater stability at pH ≥ 7.0. However, acetogenins were reduced by 63 and 32 % at 25 and 4 °C for 42 days. Among acetogenins, persediene was the most stable, followed bypersenones andAcO-avocadene with an aliphatic chain, a keto group, or *trans*-enone in C-4 allow hydrogen donation to a carbon atom and inhibit oxidation (Pacheco et al., 2017).

Villarreal-Lara et al. (2019) conducted a study to evaluate the anti-microbial spectrum of avocado seeds acetogenins. They added purified acetogenins meat inoculated with *Listeria monocytogenes* and then stored at 20 and 4 °C. They exposed eight gram-positive bacteria to Nisaplin® and Mirenat® (food preservatives) for comparative analysis. The authors concluded that the inhibition zone of avocado seeds acetogenins was two-four times higher than Nisaplin® and Mirenat® for gram + ve bacteria, except for *Staphylococcus aureus*. Additionally, after storage at 4 °C for 72 d, acetogenins inhibited *L. monocytogenes* completely. These suggested avocado seeds as a good source of functional compounds with anti-microbial potential.

Chemical profiling of volatile compounds indicated that avocado seed contains sesquiterpenoids, poly, and unsaturated fatty acid esters (Soledad et al., 2021). They reported that the fatty acids of avocado seeds display antimicrobial activities. The fatty acid has double bonding in the *cis*-configuration. They alter its functionality by disordering the phospholipid chain, resulting in fluidity, disorganization, and disintegration of the cell membrane resulting in leakage of intracellular content and cell death. A high concentration of 2000 mg/L exhibited maximum microbial reductions of 4 and 1.8 log cycles for *Staphylococcus aureus* and Salmonella* enterica* serovar typhimurium, respectively (Soledad et al., 2021). They explained that minimum microbial reduction for S. *typhimurium* could be due to the composition and cell wall structure. Gram-negative bacteria exhibit lipidic bilayer providing more protection against antimicrobial components (Beristain-Bauza et al., 2019).

### Valorization approaches in the food industry

The industrial processing of avocados generates various by-products such as peel and seed, in which seed is a major waste product that accounts for about 13 % to 17 % of avocados. The avocado seed is ideal for valorization because it includes various nutritious components with numerous potential industrial uses. Its seed powder and flour have many specific nutrient contents, which encourage scientists to work on the utilization of seed in various food products, i.e., Instant soup and beverages (Alissa, Hung, Hou, Lim, & Ciou, 2020), antioxidant-rich tea (Araujo et al., 2018); antibacterial agent in meat products (Villarreal Lara et al., 2019), antioxidant in sunflower oil (Segovia et al., 2018), used as a preservative (Pachego et al., 2017), and bakery products (Rivera Gonzalez et al., 2019). Further development and commercialization of these research efforts by the food industry will provide an opportunity for a raw material source that is still underutilized and generally treated as waste.

### Application in functional food formulations

**Fig. 2:**
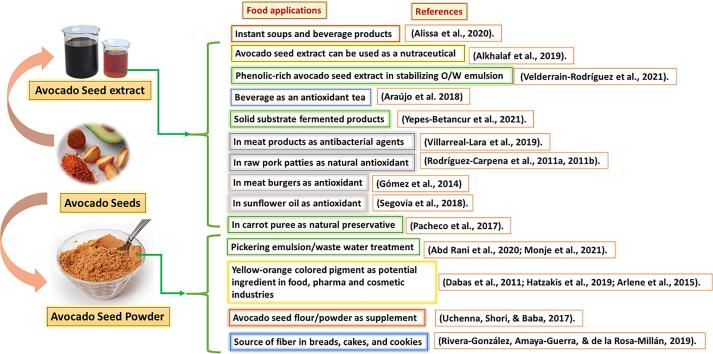
Various food applications of avocado seed powder and extracts.

The avocado seed contains various classes of nutritional components (carbohydrate, protein, olefinic and acetylenic bond containing fatty acids, fiber, and minerals) as summarized in section 2 and other natural products such as phytosterols, triterpenes, dimmers of flavonols, and oligomeric pro-anthocyanidins (discussed in section 3) which can be explored in designing of different functional foods to stimulate growth and metabolism (Permal, Chang, Seale, Hamid, & Kam, 2020). The up-to-date food applications of avocado seeds (flour and extract) have been summarized in Fig. 2. When included in the diets, avocado seed flour showed dose-dependent partial effects on the feeding and growth performance of rats (Uchenna et al., 2017). The cholesterol levels were lowered; high blood glucose was suppressed, especially after adding sucrose to the diet. The liver glycogen storage of rats improved after avocado seed inclusion in diets. Therefore, avocado seed flour can modulate lipid and carbohydrate metabolism and improve the glycogen storage ability of the liver and can be utilized in the diets of people with hyperglycemia and/or hypercholesterolemia. Due to the seeds' dietary and crude fibre, antioxidants, and phenolic content, Pahua-Ramos et al. (2012) also discovered minimal toxicity, hypocholesterolemia, and low LDL cholesterol in hypercholesterolemic model mice. The avocado seed powder-supplemented diets of culled female quail improved its kidney and liver functions, meat quality, tenderness, protein, and fat content, while cooking losses were reduced (Tugiyanti, Iriyanti, & Apriyanto, 2019).

A vegetable extract prepared from avocado and soybean fruit and seed oil (mainly in the ratio of 1:2) is known as avocado and soybean unsaponifiables (ASU). It has anti-inflammatory effects attributed to many phytosterols and isoflavones, which suggests its possible role in the prevention of osteoarticular, autoimmune, and menopausal disorders (Eser et al., 2011). ASU as a pure extract or mixed with other plant extracts (e.g., *Uncaria tormentosa* and *Zingiber offcinalis*) is available as food supplements in many countries (Ghasemian et al., 2016, Salehi et al., 2020). The seed extract can be used as a nutraceutical due to its antioxidant, anti-inflammatory, and antibacterial potential, having more powerful effects than avocado bulb extracts (Alkhalaf et al., 2019). In an indomethacin-induced ulcer study in mice, the ethyl acetate fraction of avocado seed extract was found effective in decreasing the level of oxidized products and increased superoxide dismutase enzyme activity, thus mitigated the oxidative stress and also prevented the increase in the ulcer and lesions (Athaydes et al., 2019). The extract is rich in substances such as flavonoids, epicatechin, catechin, caffeoylquinic acid, phenylpropanoids, and tannins, proving it a valuable nutraceutical that can be used a safe, effective, and cheap alternative to conventional treatments to prevent or treat gastric ulcers. However, further studies and clinical assays are required to develop more scientific information and one formulation for sustainable utilization (Athaydes et al., 2019).

To improve the nutritional quality and antioxidant profile of different seeds, solid substrate fermentation has been focused on these days (Dhull et al., 2020, Dhull et al., 2021). Solid substrate fermentation of avocado seeds using fungi such as *Aspergillus niger* resulted in the secretion of bound phenolic compounds and increased antioxidant capacity. Several factors such as low cost, fast growth rate of microbes, easy downstream of phenolic/fermentation compounds and eco-friendly nature have proven fermentation as an efficient process for the production of polyphenolic compounds. Meanwhile, different enzymes (protease, amylase, lipase, phytase etc.) produced by fermenting microbes convert complex carbohydrates, proteins and lipids into easily digestible components with an appealing taste and texture (Dhull et al., 2020, Dhull et al., 2021). Also, these enzyme significantly reduce different anti-nutritional factors, including tannins, phytic acid, and protease inhibitors (Soetan & Oyewole, 2009), and help to improve the absorption and bioavailability of certain minerals present in seeds. This suggested an opportunity to increase the value of processing avocado waste and develop new products to avoid processing waste (Yepes-Betancur et al., 2021). A seed powder prepared by spray drying a mixture of avocado seed extract, maltodextrin, and water showed good yield (24.46 %–35.47 %), water activity (0.27 %–0.34 %), solubility (55.50 %–79.67 %), and color values (Alissa et al., 2020). This powder can be used in different food such as instant soups and beverage products, simultaneously adding value to the waste product.

The growing trend towards no or minimal use of synthetic additives forced the food industries to use natural additives and discover new antimicrobial molecules (Tiwari et al., 2009; Negi, 2012). Avocado seed extract is a rich source of acetogenins which have strong antimicrobial, antifungal, and insecticidal properties (Pacheco et al., 2017; Salinas- Salazar et al., 2017; Villarreal-Lara et al., 2019, Salazar-López et al., 2020). The seeds are almost 1.6 times richer source of acetogenins than the pulp, showing a good waste management solution for the avocado processing industry (Salinas- Salazar et al., 2017). The extract inhibited *Listeria monocytogenes* completely and showed antibacterial activities against several Gram-positive bacteria, including *Bacillus subtilis, Staphylococcus aureus, Clostridium perfringens*, *C. sporogenes,* and *Alicyclobacillus acidocaldarius* (Villarreal-Lara et al., 2019). The acetogenins from avocado seeds were characterized for the anticlostridial activity, stability and effectiveness under different food processing conditions and in a model food system (Pacheco et al., 2017). The extract bioactivity showed resistance to different food processing conditions such as HPP (300 MPa –600 MPa, 3–6 min, 25 °C), high temperature (≤120 °C), and salt concentration (≤3 % w/v), the extract had good resistance while showed higher stability at pH ≥ 7.0. Additionally, after exposure to HHP treatment and pH 9.5, an increase in the potency against endospores was observed, suggesting a positive effect on the solubility or structure of particular acetogenins. However, the initial quantity of acetogenins was gradually decreased in HHP processed model food system (carrot puree) during storage at different temperatures. The antioxidant activity conferred by hydrogen donation to surrounding carbon atoms could be due to a keto or *trans*-enone group at C-4 in the aliphatic chain of the acetogenins. This suggested the potential of avocado seeds as natural food preservatives, but further investigation regarding the effectiveness of acetogenins against different microorganisms, its stability in more complex food systems, effect on sensory attributes, and human consumption safety evaluation is needed. In combination with nisin (an antimicrobial peptide), the seed extract acted synergistically in its microbial response, providing a novel combination to decrease nisin use at the industrial level, reducing cost, and promoting the utilization of natural resources compounds (Calderón-Oliver et al., 2016).

In lipids and protein-rich foods, lipid oxidation and protein carbonylation lead to nutritional loss, off-flavors, loss of essential amino acids, reduced digestibility of myofibrillar protein, and degradation of their texture and other quality traits (Shahidi & Zhong, 2010). Avocado seed extracts are an interesting natural source of rich phenolic compounds with strong antioxidants and antimicrobial properties. Its addition to the meat system would enhance nutritional and sensory properties by effectively inhibiting the oxidation of protein and lipids. In raw pork patties, avocado seed extracts reduced oxidative reactions and color deterioration during storage through protein carbonyl formation and TBARS reduction (Rodríguez-Carpena et al., 2011, Rodríguez-Carpena et al., 2011).

In emulsion-based foods, lipids are present in dispersed colloidal particles (O/W emulsions) and stabilized by surface-active compounds, including proteins, polysaccharides, gums which act at the interfacial regions (Decker et al., 2017). As the molecules at the interface come in contact with many pro-oxidants (enzymes, metals, photosensitizers, etc.), the oxidative and colloidal stability of emulsions highly depends on the composition of the interface (Yi et al., 2019). In the O/W emulsion, lipid oxidation results in the development of off-flavors, and shortening of shelf-life, which causes rejection by consumers and influences food safety by forming toxic reaction products. The phenolic-rich avocado seed extract significantly affect the colloidal stability of O/W emulsions, depending on the emulsifier used to prepare the emulsion/nanoemulsion (Velderrain-Rodríguez, Salvia-Trujillo, González-Aguilar, & Martín-Belloso, 2021). Also, the lipid oxidative stability of emulsions and nanoemulsions was enhanced as the phenolic components in the extract retarded the oxidation process. It decreased the formation of secondary lipid oxidation products during storage. The extract from avocado seeds also inhibited oxidation of about 30 % in pure form and about 60 % in combination with egg albumin. In comparison, TBARS formation was reduced by 90 % in meat burgers (Gómez et al., 2014), suggesting its possible use as an antioxidant in foods. The oxidation was also delayed in sunflower oil with added avocado seed extracts (Segovia et al., 2018) which suggested its use in reducing synthetic surfactants and additives.

Avocado seed processed into flour have good yield (46.3 %), protein (6.7 %), fat (3.4 %), ash (2.7 %), and dietary fibre (45.53 %) contents which can be an alternative source of nutrients for the preparation of bread, cakes, and cookies (Rivera-González et al., 2019). The flour has good water and oil absorption capacity and solubility (2.4 %, 2.16 %, 11.2 %, respectively) related to different macro and micronutrients present in the seeds and their strong intermolecular interactions. Still, additional compounds such as gum, pectin, alginates can be added to avocado flour to alter its properties for better exploration. Avocado seeds are a rich source of dietary fiber which has many health benefits such as hypoglycemia, hypocholesterolemia (Hu & Yu, 2013), cardioprotective and prebiotic (Slavin, 2013), early satiety (Kristensen & Jensen, 2011), and excretion and retention of bile juices (Kristensen et al., 2012). The avocado seeds fibrous residue has useful technological properties, including good water and oil absorption properties, suggesting its use as an important ingredient to improve softness, freshness, and viscosity in bakery products and juiciness in meat products (Barbosa-Martín, Chel-Guerrero, González-Mondragón, & Betancur-Ancona, 2016). The seed also contains fatty acids esters, and unsaturated fatty acids, which are beneficial for human health; therefore, suggesting the use of these solvent-extracted compounds in developing functional foods (Soledad et al., 2021). Natural anti-diabetic and flavonoid-rich substances from avocado seeds can regulate blood glucose levels in many ways (Brahmachari, 2011), which can be explored in the preparation of alternative snacks such as biscuits used for diabetes management (Mursyid & Kadir, 2020).

The avocado seeds can also be utilized as alternative/non-conventional starch sources (Rivera-González et al., 2019). Its starch fraction (27.3 % yield) have low total dietary fibers (7.32 %) (Rivera-González et al., 2019), which can be useful as a thickener, emulsifier, and gelling agent in different food applications (Dhull et al., 2022, Chandak et al., 2022, Chandak et al., 2022). The avocado starch contains 15–16 % amylose, with a gelation range at 56–74 °C having good water absorption capacity (22–24 g of water/g of starch), solubility (19 %–20 %), swelling power (28 g–30 g of water/g of starch), and a maximum viscosity (380 BU–390 BU) which makes it an ideal ingredient for gelling and thickening, pharmaceuticals and biodegradable packaging materials for foods (Chel-Guerrero, Barbosa-Martín, Martínez-Antonio, González-Mondragón, & Betancur- Ancona, 2016). Further, treatment like microwave-assisted extraction of avocado seed starch has been reported improving the extraction yield and induce some changes in the structure and properties of starch, for example, the production of small size starch chain and improving the starch solubility (Araújo et al., 2020). These changes can improve the starch functionality and provide new biotechnological applications, such as formulation of nanoparticles and preparation of oligomers with bioactivity (Araújo et al., 2020). Starch has a huge world of utilization, which can be further extended by many modifications such as oxidation of avocado seed starch with standard sodium hypochlorite solutions (Lacerda et al., 2014) and heat moisture treatment (Lacerda et al., 2015). These modifications change various physicochemical properties of starches, such as average roughness, gelatinization enthalpy, pasting properties, degree of relative crystallinity, making them more suitable for several food industry applications (Punia et al., 2019, Punia et al., 2020, Dhull et al., 2020c). Alrefai et al. (2020) prepared bioplastic sheets with optimal properties and cost by mixing potato starch (47 g), mango starch (7.6 g), and avocado starch (35.3 g), opening more ways for utilization of avocado seeds.

Recently, avocado seeds demonstrated their utilization as a bio platform for producing relatively new nanomaterials, i.e., carbon dots (CD) with polyfunctional surfaces and different physico-chemical properties (Monje et al., 2021). Apart from its several applications in other fields, the CD can be effectively used as a Pickering emulsion (i.e., an emulsion stabilized by solid surfactant) stabilizer (Zhai et al., 2018) due to its high dispersibility in water. The CD can be used as a solid surfactant to avoid the adverse effect of soluble surfactants, affecting human health and the environment due to their mutagenic, toxic and carcinogenic properties (Chevalier & Bolzinger, 2013). Further, using a nanomaterial like CD with all dimensions less than 10 nm would help prepare emulsion with very small droplet sizes (Zhai et al., 2018), which may find its utilization in food drug delivery, and cosmetics. Copper nanoparticles ranging from 42 to 90 nm synthesized using a green route with avocado seed extract were found stable and reproducible with excellent antioxidant and antimicrobial properties against the plant pathogens (*A. niger, A. fumigatus, F. oxysporum*) (Rajeshkumar & Rinitha, 2018). These bio-medically important nanoparticles can be utilized in drug delivery, nutraceuticals, and other food and pharma applications.

Apart from this, distinct photoluminescent properties and singlet oxygen photosensitizing capacity of the CD is of interest in wastewater treatment and catalysis (Abd Rani et al., 2020, Monje et al., 2021). Similarly, different avocado seed based adsorbents find several applications in wastewater treatment by removing basic dyes (Elizalde-González, Mattusch, Peláez-Cid, & Wennrich, 2007), phenol (Rodrigues, da Silva, Alvarez-Mendes, dos Reis Coutinho, & Thim, 2011), ammonium, and p-cresol (Zhu et al., 2016, Zhu et al., 2018), fluoride (Salomón-Negrete, Reynel-Ávila, Mendoza-Castillo, Bonilla-Petriciolet, & Duran-Valle, 2018), organic pollutants including pharmaceuticals and phenols (Leite et al., 2018), methylene blue dye (Dhaouadi et al., 2020), anticancer drug (Della-Flora et al., 2020), and heavy metals (Boeykens et al., 2019, Dhaouadi et al., 2021, Díaz-Muñoz et al., 2016).

Moreover, a yellow-orange-colored pigment extracted from avocado seeds (Dabas et al., 2011, Hatzakis et al., 2019) can also be a potential food ingredient (Arlene, Prima, Utama, & Anggraini, 2015), pharma and cosmetic industries but after its further safety assessment studies. Eighteen patents related to avocado have been reported by Araújo, Rodriguez-Jasso, Ruiz, Pintado, and Aguilar (2018). The majority (i.e., ten) of that is related to the food industry, such as using avocado seed as a tea ingredient or a drink and as a substance to prepare culture media. Besides food, nutraceutical, pharma, and feed applications, avocado seeds may be important for personal care. Avocado seed extract flavonoids and secondary metabolite, such as catechin, can inhibit the process of melanogenesis and have skin lightening potential by inhibiting the tyrosinase activity (Laksmiani, Sanjaya, & Leliqia, 2020). Also, four patents related to cosmetic, including one for an avocado facial cleanser, stands out for avocado-related products (Araújo et al., 2018, Salazar-López et al., 2020). This could demonstrate the diverse and effective use of avocado seeds, reducing contamination by not ending up as waste and generating nutritional and health benefits and economic gains.

## Conclusion and future prospects

Avocado is widely grown and consumed fruit crop in tropical and subtropical regions while exported to the rest of the world because of its delicious taste, rich nutrient composition, and several health-promoting bioactivities in the human system. However, its seeds are generally considered as agricultural and food processing waste. The present review article has discussed that the seed remnants generated from the avocado fruit processing industries also exhibit several important constituents such as proteins, polyphenolic compounds, unsaturated fatty acids, antimicrobials and polysaccharides with promising biological and functional properties. The *in vitro* and *in vivo* studies on animal models along human cell lines using avocado seed extracts have proved its health-promoting properties like a strong antioxidant, anti-microbial, anticancer, anti-obesity, anti-inflammation, anti-diabetes, and anti-neurogenerative agent. Based on the nutritional and healthful bioactivities of avocado seeds, the present review also summarized their applications in the development of functional food for cancer and diabetic patients. Several experiments have been performed to validate the safety of avocado seed consumption; however, reports on its mechanism of action and metabolism in humans are limited. It is evident that a multidisciplinary research approach has encouraged the utilization of avocado seed residue as a healthy ingredient in the food industry. Recently, the application of avocado seed in functional food or food ingredients has gained much attention from many researchers. However, the understanding is still unearthed with respect to the molecular mechanism of bioactivities of avocado seed extracts. As the food industry looks to become more sustainable, repurpose of waste generated during processing into value-added products is essential. An in-depth investigation on the safety and pharmacological activities of specific compounds from avocado seed extract for pharma and food application needs to be proved. Also, there needs to be further research into valorization approaches as a cost-effective raw material.

## Funding

Supported by a grant from the Romanian National Authority for Scientific Research and Innovation, CNCS—UEFISCDI, project number PN-III-P2-2.1-PED-2019–1723 and PFE 14, within PNCDI III.

## Declaration of Competing Interest

The authors declare that they have no known competing financial interests or personal relationships that could have appeared to influence the work reported in this paper.

## Data Availability

The authors do not have permission to share data.
